# Comparison of the Performance of Urinary* Mycobacterium tuberculosis* Antigens Cocktail (ESAT6, CFP10, and MPT64) with Culture and Microscopy in Pulmonary Tuberculosis Patients

**DOI:** 10.1155/2017/3259329

**Published:** 2017-10-18

**Authors:** Dewi Kartika Turbawaty, Adhi Kristianto Sugianli, Arto Yuwono Soeroto, Budi Setiabudiawan, Ida Parwati

**Affiliations:** ^1^Department of Clinical Pathology, Faculty of Medicine, Universitas Padjadjaran, Dr. Hasan Sadikin General Hospital, Bandung, Indonesia; ^2^Department of Internal Medicine, Faculty of Medicine, Universitas Padjadjaran, Dr. Hasan Sadikin General Hospital, Bandung, Indonesia; ^3^Department of Pediatrics, Faculty of Medicine, Universitas Padjadjaran, Dr. Hasan Sadikin General Hospital, Bandung, Indonesia

## Abstract

Pulmonary tuberculosis (TB) is a major global health problem and is one of the top 10 causes of death worldwide. Our study aimed to evaluate the performance of urinary* Mycobacterium tuberculosis *(Mtb) antigens cocktail (ESAT6, CFP10, and MPT64) compared with culture and microscopy. This descriptive cross-sectional study was conducted in Dr. Hasan Sadikin General Hospital, Bandung, from January 2014 to October 2016. A total of 141 pulmonary tuberculosis patients were included. Sputum samples were examined for acid-fast bacilli (ZN stain) and mycobacterial culture (LJ); the Mtb antigens cocktail was examined in the urine sample. The positivity rate of TB detection from the three methods was as follows: AFB 52/141 (36.9%), culture 50/141 (35.5%), and urinary Mtb antigens cocktail 95/141 (67.4%). Sensitivity, specificity, PPV, and NPV of urinary Mtb antigens cocktail were 68.2%, 33%, 31.6%, and 69.6%, respectively. Validity of combination of both methods with culture as a gold standard yielded sensitivity, specificity, PPV, and NPV of 90%, 28.6%, 40.9%, and 83.8%, respectively. Combination of urinary Mtb antigens cocktail with AFB as a screening test gives a good sensitivity, although the specificity is reduced. Urinary Mtb antigens cocktail can be used as screening test for pulmonary tuberculosis.

## 1. Introduction

Tuberculosis (TB) is a major global health problem. It causes ill health among millions of people each year and is one of the top 10 causes of death worldwide [1]. Recently, it is estimated that there are about 1 million new TB cases per year in Indonesia, which is twice greater than the previously estimated period [2].

In 2015, only 6.1 million people had access to quality TB care and 4.3 million people were missed out; therefore better diagnosis will close this gap. The diagnosis of pulmonary tuberculosis is based mainly on sputum smear microscopy and/or mycobacterial culture [3]. A microscopic investigation using acid-fast bacilli (AFB) stain is the primary laboratory tool for* Mycobacterium* species, which is inexpensive, fast, and specific [4]. Difficulties of* Mycobacterium* species detection are as follows: (1) the sensitivity of the sputum microscopy is 20–60%; this test has high specificity but some mycobacteria other than* M. tuberculosis* may also stain acid [5]; (2) the result of culture technique requires 2-3 weeks, and positivity of the culture depends on number of viable bacteria. Therefore, it may cause delay to provide a definitive diagnosis for tuberculosis [6]. Recently, new diagnostic tools for* Mycobacterium* detection have been published, for example, whole blood interferon gamma (IFN-*γ*) release assays (IGRAs) and real-time polymerase chain reaction- (PCR-) based GeneXpert. The new diagnostic tools seem unsuitable for routine clinical use, especially for poor resource settings, where the majority of TB cases occur. Moreover, those new tools require sophisticated laboratory setups, equipment, and trained personnel [7–10].

The mycobacterial antigens secreted (ESAT6, CFP10, and MPT64) give an opportunity to be part of TB diagnostic strategy. The antigens were encoded by the genes of a region of difference 1 (RD1) and RD2 [11, 12]. The 10 kDa culture filtrate protein (CFP10) and 6 kDa early secreted target antigen (ESAT-6) are two low molecular weight secretory proteins which are encoded by the Rv3874 and Rv3875 gene, respectively. These genes are located in the RD1 of the Mtb genome but are absent in all* M. bovis* bacillus Calmette-Guerin (BCG) vaccine strains [13]. Therefore, CFP10 and ESAT-6 play an important role for mycobacterial virulence and pathogenesis [14, 15]. The tuberculosis 28 kDa antigen MPT64, also termed as protein Rv1980c, is located in the RD2 of the* M. tuberculosis* genome. This protein is secreted by actively growing* M. tuberculosis* strains. The previous study has proven that MPT64 antigen is only found on viable and actively dividing cells of* M. tuberculosis *[16].

The detection of Mtb specific antigens has been an important diagnostic aid in the diagnosis of TB. Currently, the secreted Mtb specific antigens (ESAT6, CFP10, and MPT64) have been evaluated for their serological diagnostic potential. Previous studies have described immunodiagnostic tests for tuberculosis based upon the detection of Mtb antigens using cerebrospinal fluid and sputum samples [3, 17, 18]. As the tests are highly sensitive and specific, they improve the positive diagnostic rate. Further, several research groups reported that a combination of multiple antigens could improve the diagnosis of pulmonary TB [3, 10, 19–22].

Urine is an ideal clinical specimen because it is excreted in large quantities and the collecting process does not require invasive methods. Normally, the kidney will excrete urinary antigens with low molecular weight (<67 kDa) or transrenal DNA that does not exceed 100 bp. However, only a few studies evaluated the performance of a cocktail of Mtb antigens ESAT-6, CFP-10, and MPT-64 from urinary samples. It is expected that the detection of direct excreted antigens in urine could become a potential candidate diagnostic tool for Mtb when conventional culture remains negative or unreliable [23, 24]. The aim of this study was to evaluate the performance of urinary Mtb antigens cocktail (ESAT6, CFP10, and MPT64) in pulmonary tuberculosis subjects.

## 2. Materials and Methods

### 2.1. Study Population

This study was conducted between January 2014 and October 2016 at Dr. Hasan Sadikin General Hospital, Bandung, Indonesia, as tertiary referral hospital in West Java province. The patients were screened in a TB outpatient clinic. The inclusion criteria were patients with unexplained productive cough lasting two-three weeks or more, often accompanied by systemic symptoms, such as fever, night sweats, and weight loss, according to International Standard of Tuberculosis (ISTC), and chest radiography findings suggestive of tuberculosis [25].

### 2.2. Specimen Collection

This study required three consecutive sputum samples and the mid-stream urine, which were collected per patient prior to initiation of TB treatment. The sputum and the mid-stream urine were collected into a clean sterile container. Both male and female participants were given instructions on proper specimen collection by the study staff.

### 2.3. Laboratory Procedure

Direct smears were prepared from each sputum specimen for microscopic investigation using AFB stain by the Ziehl Neelsen (ZN) technique (ST Reagensia Company, Jakarta, Indonesia) and interpreted according to standard guidelines. The remaining sputum specimens were decontaminated by a standard N-acetyl-L-cysteine- (NaLC-) NaOH method and concentrated with centrifugation at 3000*g* for 15 minutes. The concentrated sputum was used for culture. Of those who had provided ≥1 specimen, the sputum specimens were mixed before decontamination process. The Lowenstein-Jensen (LJ) agar (Biomerieux S. A, Marcy l'Etoile, France) and TB Ag MPT64 rapid tests (SD Diagnostic, Korea) were performed to identify Mtb. Observation of TB culture was performed every week from 3 to 8 weeks. The LJ agar was considered positive if the mycobacterial growth was more than one colony forming unit (CFU). Three laboratory staff members were participated in proficiency testing prior to enroll the clinical specimens.

### 2.4. Urinary Mtb Antigens Cocktail

Mtb antigens cocktail in urine samples is detected using the TB antigen cocktail rapid immunochromatography test (ICT) (Jei Daniel Biotech Corp., Taiwan). One hundred *μ*L of sample buffer was taken into the specimen collection box and added with 100 *μ*L of urine sediment. It was then mixed well by dropper, up to down in 30–60 seconds then to stand for 30 minutes. Four drops (60 uL) were applied into “S” region of the card for testing. The test result was read after 30 minutes. The result was considered positive if distinct pink colored band appeared in control region and test region. It was considered negative if only one pink colored band appeared on the control region with no apparent band on the test region. The test result was considered invalid when there was no pink colored band in both regions, indicating procedure errors and/or test reagent deterioration.

### 2.5. Data Analysis

The AFB stain and Mtb antigen cocktail results were collected and tabulated, against mycobacterial culture results as the gold standard. The sensitivity, specificity, positive predictive value (PPV), and negative predictive values (NPV) were presented for AFB stain, Mtb antigens cocktail, and a combination of both. The data were analyzed using statistical software. The results of the study were presented as diagram figure and table with number and percentage.

### 2.6. Ethics

This study was approved by the Ethical Committee of the Faculty of Medicine, Universitas Padjadjaran, and Dr. Hasan Sadikin General Hospital (number 493/UN6.C1.3.2/KEPK/PN/2014). Written informed consent was obtained from all participants.

## 3. Results

**Figure 1:**
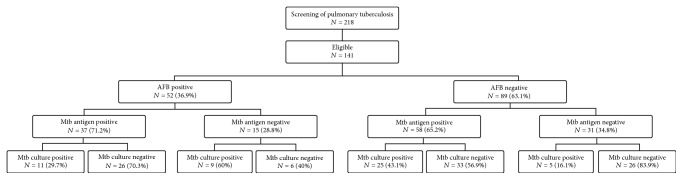
The pulmonary tuberculosis patient diagnostic workup.

From January 2014 to October 2016, 218 participants were enrolled. Of those, 77 participants were excluded because they failed to give consent or urine specimens. The 141 study participants had a median age of 35 (23–47) years; 91 (64.5%) were male. The majority of the participants had normal body mass index (53.1%) (Table 1). The positivity of urinary Mtb antigens cocktail was 67.4% (95/141), greater than mycobacterial culture and AFB (Figure 1). The combination of sputum AFB smear with urinary Mtb antigens cocktail against mycobacterial culture as gold standard gave the sensitivity and NPV as high as 90% and 83.8%, respectively (Table 2). For AFB smear-negative case, the addition of urinary Mtb antigens cocktail could increase the positivity for 43.1%, compared with mycobacterial culture (Figure 1).

## 4. Discussion

Microscopic investigation of sputum is still the most commonly used method for tuberculosis diagnosis. Although the microscopic investigation is the fastest diagnostic method, the performance of sensitivity is relatively low. For a positive result, samples must contain more than 10.000 bacilli per milliliter [5]. This is shown in our study for the performance of AFB stain against mycobacterial culture, as high as 45.4% for sensitivity and 67% for specificity. Recently, the detection of Mtb specific antigens has been an important diagnostic which aid in the diagnosis of TB. The mycobacterial antigens secreted (ESAT6, CFP10, and MPT64) have been evaluated for their serological diagnostic potential. Our study was the first study evaluating the presence of antigens secreted by Mtb specific antigens (ESAT6, CFP10, and MPT64) in urine. We demonstrate the performance between microscopic investigation and Mtb antigens cocktail, with mycobacterial culture as a gold standard in TB patients (Table 2). Our finding showed that the combination of Mtb antigens cocktail helps the detection case of pulmonary TB. This finding is similar with previous study, which described the immunodiagnostic tests for tuberculosis based upon detection of* Mycobacterium tuberculosis *antigens and claimed high sensitivity that could improve the positive diagnostic rate of pulmonary TB [3, 10, 19–22, 26, 27].

During this study, we found false positive and false negative for Mtb antigens cocktail (Table 2), tested either as single test or as combination test, against mycobacterial culture. Some aspects can contribute to the high number of false positive Mtb antigens cocktail. First, mycobacterial culture is still the reference standard method for pulmonary tuberculosis diagnosis. The culture needs the presence of 10–100 bacilli in a milliliter of the sample to obtain the culture sensitivity of 81.5% and the specificity of 98.4% [5]. This shows that culture positivity depends on the number of bacilli in a milliliter of sample. Second, a positive culture was affected by the viability of* M. tuberculosis*, without good specimen handling or long transportation of sputum sample may cause death of bacteria, which cannot grow in media culture. Third, numbers of studies suggest that ESAT6 and CFP 10 have been linked to cell lysis of both macrophages and pneumocytes. They also suggested that ESAT6 could induce pore formation on the macrophage and dendritic cell membranes, resulting in the spreading from intracellular, independently from bacterial load [15, 28]. Therefore, those secreted antigens can be found in the urine although* M. tuberculosis* was still inside the macrophage. Similar to tuberculosis lipoarabinomannan (LAM), we observe the false negative of Mtb antigens cocktail may be impacted by variable concentration of the urine sample [29].

During the study, we also observed the AFB smear-negative patients. The patients contributed for 63.1% among the study participant, with positive* Mycobacterium* culture of 27%. By adding the urinary Mtb antigens cocktail, our study showed the additional performance from this test as screening, which could increase the case detection up to 43.1%. This finding proves the sensitivity and NPV performance of urinary Mtb antigens cocktail.

The limitation of this study is the different levels of education of the subjects. This may influence the understanding on how to collect proper sputum and mid-stream urine, although study staff had taught all subjects. This could influence sputum examination and urine TB antigen cocktail results. Another limitation of our study was only using sputum culture as the gold standard. Since obtaining successful sputum culture requires number of bacteria, the gap for this limitation could be improved by using PCR-based GeneXpert, as the gold standard, which may provide a better estimate of the specificity of the AFB and Mtb antigens cocktail tests [30].

In conclusion, our study has shown the additional value of urinary Mtb antigens cocktail to improve pulmonary TB diagnosis especially in peripheral health settings. Further study is needed to get the better estimation for the specificity, and study algorithm that combines clinical, laboratory, and radiology results should be developed and tested to have better management and treatment for TB.

## Figures and Tables

**Table 1:** Characteristics of participants.

	Number *n* = 141	Percentage (%)
Sex		
Male	91	64.5
Female	50	35.5
Age (year)		
14–25	37	26.2
26–35	43	30.5
36–45	24	17.1
46–55	25	17.7
56–65	10	7.1
>65	2	1.4
BMI category (kg/m2)		
BMI < 18.5	59	41.8
18.5 < BMI < 24.99	75	53.1
BMI ≥ 25	7	4.9
Sign and symptoms of TB		
A current cough ≥ 2 weeks	141	100
Fever	84	59
Weight loss	100	71
Chest pain	98	69
Night sweats	61	43
Shortness of breath	68	48
Diagnostic Test		
AFB smear positive	52	36.9
Mycobacterial culture positive	50	35.5
HIV status positive	27	19.2

**Table 2:** Sensitivity – specificity for AFB stain and Urinary Mtb antigens cocktail among Pulmonary Tuberculosis Patient, against Mycobacterial Culture.

Variable	Mtb culture Positive (*n*)	Mtb culture Negative (*n*)	Sn (%)	Sp (%)	PPV (%)	NPV (%)
AFB stain						
(i) Positive	20	32	45.4	67	38.5	73
(ii) Negative	24	65				
Mtb antigen cocktail						
(i) Positive	36	59	71	35	37.9	69.6
(ii) Negative	14	32				
Combined AFB *AND/OR* Cocktail						
(i) Positive	45	65	90	28.6	40.9	83.8
(ii) Negative	5	26				

*Abbreviations*. AFB, acid-fast bacilli; Mtb, *Mycobacterium tuberculosis*; Sn, sensitivity; Sp, specificity; PPV, positive predictive value; NPV, negative predictive value.
